# Electroacupuncture Pretreatment Exhibits Lung Protective and Anti-Inflammation Effects in Lipopolysaccharide-Induced Acute Lung Injury via SIRT1-Dependent Pathways

**DOI:** 10.1155/2022/2252218

**Published:** 2022-03-15

**Authors:** Dan Luo, Li Liu, Hai-ming Zhang, Yu-dian Zhou, Min-feng Zhou, Jin-xiao Li, Zhao-min Yu, Qian Tang, Shu-rui Yang, Rui Chen, Feng-xia Liang

**Affiliations:** ^1^Department of Respiratory, Wuhan No. 1 Hospital, Wuhan 430022, China; ^2^Department of Acupuncture and Moxibustion, Hubei University of Traditional Chinese Medicine, Wuhan 430061, China; ^3^Department of Pathology, Wuhan No. 1 Hospital, Wuhan 430022, China; ^4^Department of Oncology, Integrated Traditional Chinese and Western Medicine, The Central Hospital of Wuhan, Tongji Medical College, Huazhong University of Science and Technology, Wuhan 430014, China; ^5^Department of Integrated Traditional Chinese and Western Medicine, Union Hospital, Tongji Medical College, Huazhong University of Science and Technology, Wuhan 430022, China; ^6^Department of Oncology, Hubei Province Hospital of Integrated Traditional Chinese and Western Medicine, Wuhan 430015, China

## Abstract

To investigate the effect of electroacupuncture (EA) on acute lung injury (ALI), a lipopolysaccharide (LPS) induced ALI mouse model was used in this study. Before receiving intratracheal LPS instillation, mice were given EA at ST36 for 7 days as a long-term treatment or one time as a short-term treatment. Lung histopathological examination, lung injury scores, lung wet/dry (W/D) ratio, and inflammatory cytokines included proinflammation factors such as TNF-*α*, IL-1*β*, and IL-6 and anti-inflammation factors such as IL-4 and IL-10 in serum and bronchoalveolar lavage fluid (BALF) were detected at the end of experiment. The results show that EA pretreatment ameliorated the lung damage and inflammatory response by LPS. In addition, we found that SIRT1 and its deacetylation of NF-*κ*B were promoted after EA pretreatment in lung tissues. Meanwhile, the expression of angiotensin-converting enzyme 2 (ACE2) is also enhanced by EA pretreatment. Thus, the present findings suggest that EA could be a potential therapy of ALI.

## 1. Introduction

Acute lung injury (ALI) and acute respiratory distress syndrome (ARDS) are critical diseases with high morbidity and mortality [1]. Clinical statistics show that sepsis is one of the most frequent risk factors for ALI and ARDS [2]. Specific pathogens including human coronaviruses (hCoVs) infection cause uncontrolled massive inflammatory activation which is also known as a cytokine storm resulting in ALI/ARDS [3,4]. Some specific inflammatory cytokines, for example, tumor necrosis factor *α* (TNF-*α*), interleukin (IL)-1*β*, and IL-6, have been identified as biomarkers for the diagnosis and prognosis of ALI/ARDS. Lipopolysaccharide (LPS) which is a major component in Gram-negative bacteria has been widely used to induce ALI/ARDS in animal models. A short exposure of LPS like inhalation will arouse an acute inflammatory response that leads to ALI [5].

Current research studies suggest that SIRT1 plays a major role in ALI [6]. Fu et al. study indicates that SIRT1 has a lung protective function. SIRT1 activator SRT1720 ameliorated LPS-induced ALI but SIRT1 inhibitor EX527 showed the exact opposite results [7]. Other studies found that the function of SIRT1 on ALI is related to its regulation of inflammatory factor such as NF-IB [8] and TNF-*α* [9].

The potential of acupuncture to treat inflammatory diseases has been widely studied [10]. A systematic review has discussed whether acupuncture at ST36 might be useful to combat the injuries induced by sepsis [11]. In an ALI model by limb ischemia/reperfusion, electroacupuncture (EA) preconditioning at ST36 and SP6 reduces pulmonary inflammation via the TLR4/NF-*κ*B pathway [12]. Manual acupuncture (MA) at ST36 before LPS instillation also mitigates ALI and pulmonary iNOS/NO expression [13]. Our previous studies have confirmed that EA could be recognized as a feasible sirt1 promoter to modulate inflammation [14]. In this study, we were trying to investigate the therapeutic effects of EA at ST36 on LPS-induced ALI in mice via SIRT1-dependent pathways.

## 2. Methods

### 2.1. Animals

40 male C57BL/6 mice, 8 to 10 weeks old, were purchased from the Beijing Vitong Lihua Experimental Animal Technology Co., Ltd. They were housed in the SPF laboratory animal room. The animals were fed under specific pathogen-free conditions and given standard laboratory chow and water. All experiments involved in this study were approved by the Animal Experimental Committee of Tongji Medical College, Huazhong University of Science and Technology.

### 2.2. Experimental Design

**Figure 1:**
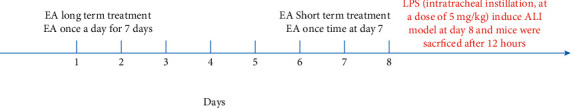
Study protocol.

Animals were randomly divided into four groups: control group (*n* = 10), LPS group (*n* = 10), LPS + EA short-term group (LPS + EA S) (*n* = 10), and LPS + EA long-term group (LPS + EA L) (*n* = 10). After one week of adaptive feeding, the LPS + EA long-term group received EA treatment, ten minutes at a time for seven days, and the LPS + EA short-term group received EA treatment for ten minutes one time before the LPS modeling. In order to simulate ALI, LPS (L2630, Sigma MO, USA) dissolved in 50 *μ*L of sterile saline was given by intratracheal instillation at a dose of 5 mg/kg for 12 h [15]. The control group received no intervention during the experiment. The study protocol was shown in Figure 1.

### 2.3. EA Delivery

Similar to our previous studies [14], the animals were immobilized using special coats and their lower limbs were exposed. Needles were applied at bilateral ST36 with a depth of 1.5 mm [13]. Needles at bilateral ST36 were inserted into one output of an electrostimulator (LH202H, HANS Electronic Apparatus), using a continuous wave of 2 Hz, 1 mA for 10 min each time.

### 2.4. Lung Wet/Dry Weight (W/D) Ratio

To assess pulmonary edema, the wet weight (W) of lung tissue was measured immediately when the mice were killed. And the tissue will be heated at 80°C for 24 h to get the dry weight (D). This data were used to calculate the ratio of W/D weight [16].

### 2.5. Haematoxylin and Eosin (HE) Staining and Immunohistochemistry

Lung tissues were fixed for 24 h in 4% paraformaldehyde. The tissue embedded with paraffin was cut into 4 *μ*m thick sections and part of the tissues were stained with haematoxylin and eosin. Other parts were incubated with monoclonal antibodies against SIRT1 (13161-1-AP, Proteintech, China) and angiotensin-converting enzyme 2 (ACE2) (ab108252, Abcam, United States) overnight at 4°C and then incubated with goat anti-rabbit secondary antibodies (Abcam, USA). 3,3′-diaminobenzidine is used as chromogen. The sections were then dyed using haematoxylin and mounted. The pathological changes of lung tissues and the expression of SIRT1 and ACE2 were observed by an optical microscope (Olympus).

### 2.6. Lung Injury Scores

Lung injury scores [17] were performed by pathologists, and the category is shown in Table 1.

### 2.7. Enzyme-Linked Immunosorbent Assay (ELISA)

We employed ELISA kits (Meilian Biotechnology, Shanghai, China) to measure TNF-*α*, IL-1*β*, and IL-6 concentrations in the serum samples and bronchoalveolar lavage fluid (BALF) samples.

### 2.8. Western Blotting

Lung tissues were homogenized in radio immunoprecipitation assay (RIPA) lysis buffer. Next, it was centrifuged at 12,000 rpm for 15 min at 4°C to get protein in the supernatants. 10% SDS-PAGE gels were used to separate proteins and then electrophoretically transfer lysates onto PVDF membranes. Membranes were blocked for 1 h with 5% nonfat dry milk and then probed overnight at 4°C with primary antibodies against Sirt1 (13161-1-AP, Proteintech, China), NF-*κ*B (10745-1-AP, Proteintech, China), ac–NF–*κ*B (ab19870, Abcam, UK), and ACE2 (ab108252, Abcam, USA), and then further incubated with an appropriate HRP-conjugated secondary antibody (Proteintech, China) for 2 h at 37°C. The densities of the bands on the membranes were scanned and analyzed by chemiluminescence and quantified with ImageJ software (Rawak Software, Inc., Germany).

### 2.9. Real-Time Reverse Transcription-Polymerase Chain Reaction

Lung tissue for RNA extraction. The RevertAid First Strand cDNA Synthesis Kit (K1622, Fermentas) was used to reverse-transcribe total RNA into cDNA. The 7900HT real-time system (7900HT Sequence Detector, ABI PRISM) and the SYBR green PCR kit (DRR081 A, TAKARA) were used. The mRNA expression was then quantified by real-time quantitative PCR (RT-qPCR). The 2−^ΔΔ^Ct method was used to express the gene expression. Specific primers used for PCR are listed in Table 2.

### 2.10. Statistical Analysis

The data in this work are presented as the mean ± standard. Differences between groups were analyzed by GraphPad Prism 7 software (La Jolla, CA, USA) with one-way analysis of variance (ANOVA) and the student Newman–Keuls test. A *P* value of less than 0.05 was considered statistically significant.

## 3. Results

### 3.1. EA Pretreatment Alleviated Lung Tissue Damage in LPS-Induced ALI

**Figure 2:**
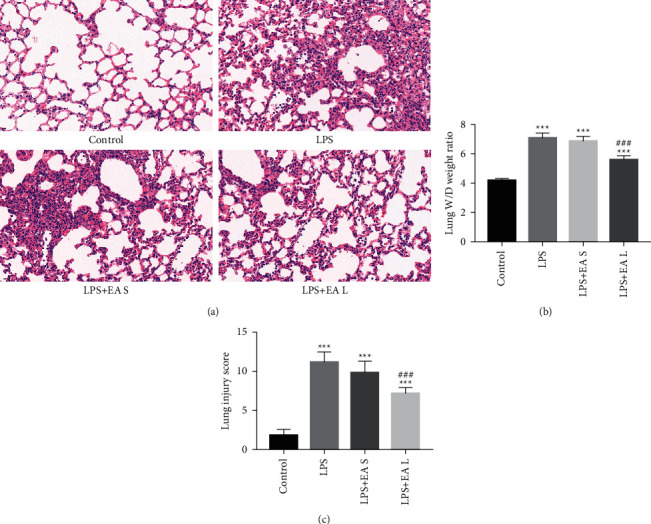
EA pretreatment alleviated lung tissue damage in LPS-induced ALI. (a) H&E staining (40×) of different groups. (b) Lung W/D ratios of different groups. (c) Lung injury score of different groups. The data are presented as the mean ± SEM. ^*∗∗∗*^*P* < 0.001 versus control group and ^###^*P* < 0.001 versus LPS group.

Pulmonary edema, epithelial and endothelial cell structure damage, inflammatory cell infiltration, and alveolar hemorrhage were observed in mice after intratracheal LPS instillation (Figure 2(a)). LPS significantly increase the lung injury score and lung W/D ratio in both LPS, the LPS + EA short-term group and the LPS + EA long term group. In the LPS + EA long-term group, pathological changes including lung injury score and lung W/D ratio were attenuated compared to those in the LPS group. Although the lung injury score and lung W/D ratio in the LPS + EA short-term group were decreased compared to the LSP group, the difference was not statistically significant (Figures 2(b) and 2(c)).

### 3.2. EA Pretreatment Attenuated Inflammatory Response in BALF and Serum in LPS-Induced ALI

**Figure 3:**
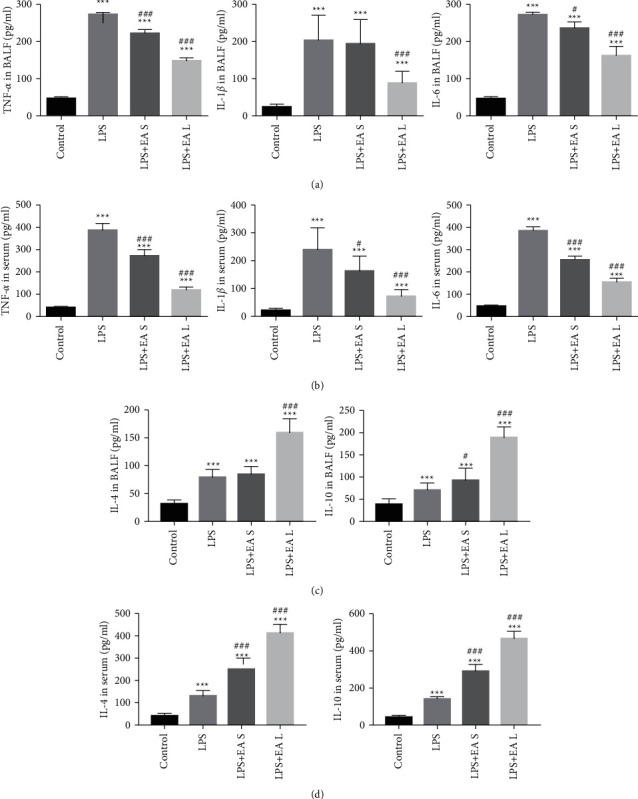
EA pretreatment attenuated inflammatory response in BALF and serum in LPS-induced ALI. (a) Proinflammatory cytokines in BALF. (b) Proinflammatory cytokines in serum. (c) Anti-inflammatory cytokines in BALF. (d) Anti-inflammatory cytokines in serum. The data are presented as the mean ± SEM. ^*∗∗∗*^*P* < 0.001 versus control group and ^#^*P* < 0.05 and ^###^*P* < 0.001 versus LPS group.

TNF-*α*, IL-1*β*, IL-4, IL-6, and IL-10 were detected in BALF and serum in both groups. After given LPS, it is observed that TNF-*α*, IL-1*β*, and IL-6 significantly enhanced. Both EA long-term and short-term pretreatment restrain the inflammatory response promoted by LPS, whereas EA short-term pretreatment has no significant effect on IL-1*β* and IL-4 in BALF (Figure 3).

### 3.3. EA Pretreatment Promoted SIRT1 Expression and Reduced the Activation of NF-*κ*B in Lung Tissues in LPS-Induced ALI

**Figure 4:**
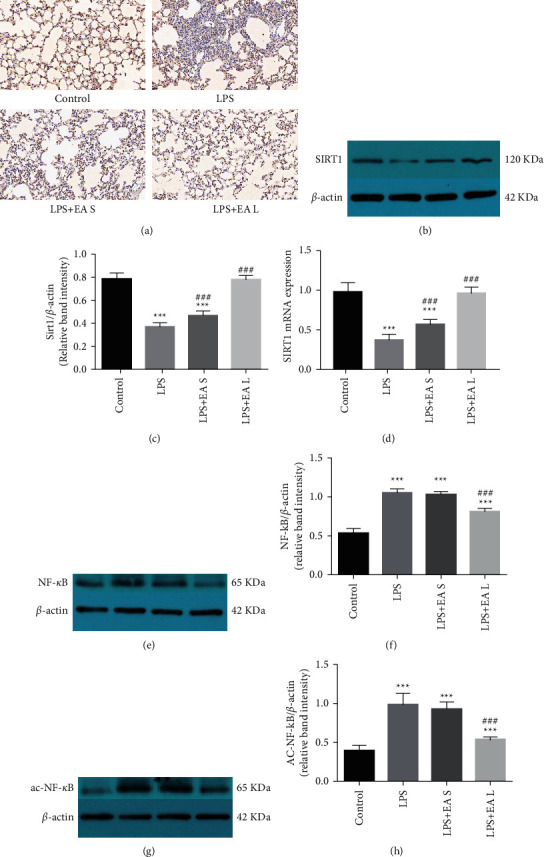
EA pretreatment promoted SIRT1 expression and reduced the activation of NF-*κ*B in lung tissues in LPS-induced ALI. (a) Immunohistochemistry analysis for SIRT1 in mouse lung tissues. (40×) (b–d) Western blot and Rt-PCR analysis for SIRT1 expression. Western blot analysis for NF-*κ*B (e, f) and ac–NF–*κ*B (g, h) expression. The data are presented as the mean ± SEM. ^*∗∗∗*^*P* < 0.001 versus control group and ^#^*P* < 0.05 and ^###^*P* < 0.001 versus LPS group.

The expression of SIRT1 in both groups was tested with immunohistochemistry (Figure 4(a)), western blot (Figures 4(b) and 4(c)), and Rt-PCR (Figure 4(d)). Both EA long-term and short-term pretreatment reversed the inhibition of SIRT1 expression in lung tissues by LPS. Our previous study showed that SIRT1 can regulate the activation of NF-*κ*B through deacetylation. In this study, the levels of both NF-*κ*B (Figures 4(e) and 4(f)) and ac–NF–*κ*B (Figure 4(g) and 4(h)) increased after exposure to LPS. EA long-term pretreatment reversed this progress, but this effect was not repeated in EA short-term pretreatment.

### 3.4. EA Pretreatment Enhanced ACE2 Expression in Lung Tissues in LP-Induced ALI

**Figure 5:**
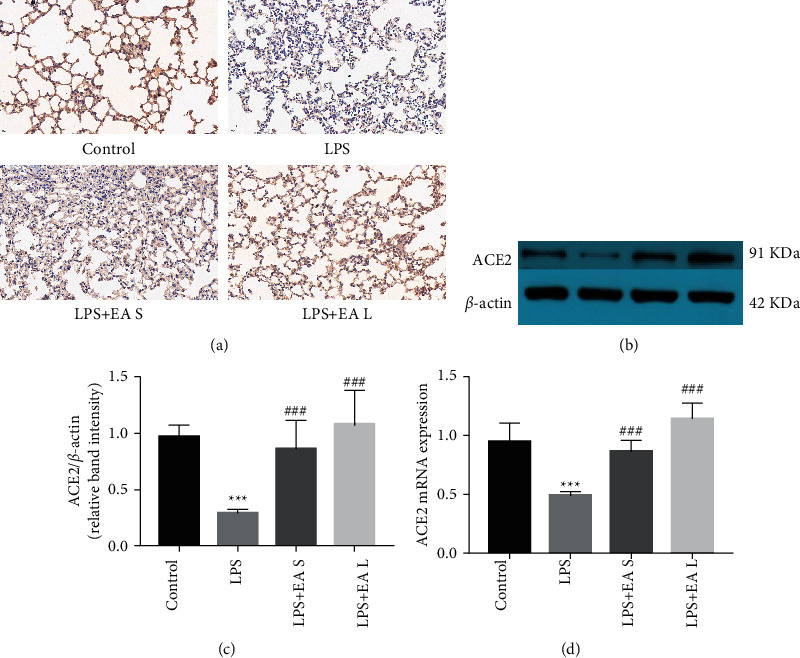
EA pretreatment enhanced ACE2 expression in lung tissues in LPS-induced ALI. (a) Immunohistochemistry analysis for ACE2 in mouse lung tissues (40×). (b–d) Western blot and Rt-PCR analysis for ACE2 expression. The data are presented as the mean ± SEM. ^*∗∗∗*^*P* < 0.001 versus control group and ^###^*P* < 0.001 versus LPS group.

ACE2 is confirmed to have a lung protective effect in ALI/ARDS [18]. To better clarify the protective effect of EA, the expression of ACE2 in each group was analyzed by immunohistochemistry (Figure 5(a)), western blot (Figures 5(b) and 5(c)), and Rt-PCR (Figure 5(d)). In ALI produced by LPS, the level of ACE2 is significantly inhibited. Both long-term and short-term EA pretreatment showed enhancement of ACE2 expression.

## 4. Discussion

In the present study, based on the LPS-induced ALI mouse model, we found that EA pre-treatment presented a lung protective effect. Lung and systemic inflammatory responses aroused by LPS were suppressed by EA. Compared with the EA short-term group, EA pretreatment at ST36 for 7 days is more effective in lung protection and anti-inflammation. The underlying mechanism of EA involves activating SIRT1 and promoting the deacetylation effect of SIRT1 to regulate NF-*κ*B. Meanwhile, the expression of ACE2 is also enhanced after EA pretreatment.

Despite significant advances in management of patients and our understanding of ALI/ARDS during the past decades, the morbidity and mortality from ALI/ARDS remains high [19]. In the pathogenesis of ALI/ARDS [20], the integrity of the capillary endothelium and alveolar epithelium was closed, which enhances edema in lung tissues and forms hyaline membranes. Activated neutrophils and macrophages secrete proinflammatory cytokines such as IL-1, IL-6, and TNF-*α*. This inflammatory process will destroy the integrity of lung tissue and increase edema.

It is widely accepted that acupuncture could be effective against inflammation in different diseases [21]. Liu et al. performed a series of studies about EA in an LPS-induced systemic inflammation mouse model. EA-evoked activation of NPY-expressing sympathetic pathways could present a quick and effective anti-inflammatory action [22]. The effect of EA at different acupoints in suppressing severe systemic inflammation could be attributed to different autonomic nervous systems [23].

There is a strong relationship between SIRT1 and pulmonary epithelial barrier dysfunction. In LPS-induced rats, upregulated expression of SIRT1 could significantly downregulate the expression of NF-*κ*B, TNF-*α*, MCP-1, IL-1*β*, MCP-1, and against cell apoptosis [24]. SIRT1 also plays a great role in maintaining the vascular integrity during ALI [7]. Hence, drugs target SIRT1 is believed to a novel therapeutic in patients with acute lung injury [8].

Increasing ACE2 activity has been recognized as a feasible approach for the treatment of ALI in the past decades [25]. ACE2 is a monocarboxypeptidase that plays an important role in maintaining the dynamic balance of the renin-angiotensin system (RAS) which attenuates immunity, inflammation, and other physiological activities [26]. There is evidence showing that SIRT1 is bound to the ACE2 promoter to enhance the expression of ACE2 under conditions of energy stress [27]. More than that, ACE2 could also prevent AT II cells from inflammatory damage via activating the SIRT1-related pathways [28].

## 5. Conclusion

In conclusion, our results showed that EA pretreatment exhibited lung protective and anti-inflammation effects in the LPS-induced ALI mouse model. The mechanisms were based on the regulation of inflammation factors through SIRT1-related pathways. Meanwhile, ACE2 may also play a key role in this progress. All the evidence supports that EA is a potential therapy to ameliorate ALI/ARDS.

## Figures and Tables

**Table 1:** Lung injury score.

Item	Score
Hyperemia	0 minimal damage to 4 maximal damage
Atelectasis	0 minimal damage to 4 maximal damage
Neutrophil infiltration	0 minimal damage to 4 maximal damage
Intraalveolar edema	0 absent to 1 present
Total	0 minimal damage to 13 maximal damage

**Table 2:** The primer sequences for SIRT1 and ACE2.

SIRT1	Fw 5′-ACGCCTTATCCTCTAGTTCCTGTG-3′
	Rw 5′-CGGTCTGTCAGCATCATCTTCC-3′
ACE2	Fw 5′-TCTGCCACCCCACAGCTT-3′
	Rw 5′-GGCTGTCAAGAAGTTGTCCATTG-3′
Actin	Fw 5′-CTATCGGCAATGAGCGGTTCC-3′
	Rw 5′-TGTGTTGGCATAGAGGTCTTTACG-3′

## Data Availability

The data used to support the findings of this study are available from the corresponding author upon request.
